# Patient blood management in Brazilian cardiovascular surgery: An exploratory survey of surgeons’ perceptions and reported practices

**DOI:** 10.1016/j.htct.2026.106500

**Published:** 2026-07-20

**Authors:** Maria Carolina Guido, Bianca Meneghini, Rosangela Monteiro, Matheus Moitinho, Carlos Manuel Brandão, Vinicius Nina, Guilherme Rabello, Fabio Jatene

**Affiliations:** a Instituto do Coração, Hospital das Clínicas HCFMUSP, Faculdade de Medicina, Universidade de São Paulo, São Paulo, SP, Brazil; b Universidade Federal do Maranhão, São Luís, MA, Brazil

**Keywords:** Patient blood management, Cardiovascular surgical procedures, Anemia, Transfusion practices, Health care disparities

## Abstract

**Introduction:**

Although the clinical benefits of patient blood management are internationally recognized, data regarding its implementation in Brazilian cardiovascular surgery remain limited. This study evaluated knowledge on patient blood management, reported practices, and perceived barriers among Brazilian cardiovascular surgeons.

**Methods:**

This cross-sectional survey was conducted among 1,105 cardiovascular surgeons registered with the Brazilian Society of Cardiovascular Surgery. A 30-item questionnaire assessing professional characteristics, preoperative anemia management, perioperative transfusion practices, and patient blood management adoption was distributed via email. Descriptive and inferential statistical analyses were performed.

**Results:**

The response rate was 19.4% (n = 205). Most respondents were male (91%) and based in the southeastern region. Preoperative anemia screening and management were heterogeneous. Thirty-one percent estimated the prevalence of preoperative anemia at 5%–15% in their institutions, and 43% reported hemoglobin testing 1–2 days before surgery. Approximately 25% routinely calculated erythrocyte mass and blood volume. Institutional transfusion risk protocols were reported by 48% of respondents, and 62% indicated standardized blood component request parameters. Antifibrinolytics and hemostatic agents were the most frequently reported coagulation strategies. Interest in patient blood management implementation was reported by 52%; perceived barriers included cultural resistance (43%), financial constraints (24%), and administrative challenges (21%). A statistically significant association was observed between geographic region and reported interest in patient blood management implementation.

**Conclusion:**

Patient blood management implementation was reported as heterogeneous across institutions. Respondents identified cultural, economic, and structural barriers to patient blood management implementation. These findings reflect surgeons’ self-reported perceptions and should be interpreted within the limitations of a cross-sectional survey with limited regional representativeness.

## Introduction

Annually, approximately two million people die from hemorrhagic causes related to trauma or surgical complications [1]. Effective blood management is therefore essential to ensure patient safety. Patient blood management (PBM) is an evidence-based strategy designed to optimize patient care and outcomes and has been recognized by the World Health Organization (WHO) as an important component of clinical practice [2,3].

PBM is a medical model that promotes the preservation and optimization of a patient’s own circulating blood, treating it with the same consideration afforded to any other organ system. It is structured around three core pillars. In the preoperative phase, the focus is on identifying and treating conditions such as anemia and coagulation disorders [4]. During surgery, the objective is to minimize blood loss through various strategies, including the use of hemostatic agents, intraoperative blood salvage, and surgical techniques that reduce bleeding [5]. Postoperatively, rigorous monitoring is performed to optimize the patient’s physiological tolerance to anemia and avoid unnecessary transfusions.

Despite its demonstrated effectiveness, PBM implementation may encounter several barriers, including limited awareness, financial constraints, and structural limitations. The transition to this care model requires cultural transformation within healthcare teams and a patient-centered approach supported by adequate institutional infrastructure. Several countries, including Australia, the United States, and members of the European Union, have implemented structured PBM programs [6,7].

Cardiovascular surgery is a major contributor to the demand for blood components, accounting for a substantial proportion of perioperative transfusions. In high-volume cardiac surgery centers, annual blood component utilization may reach 10,000 units. Although demand is lower in medium- and small-volume centers, ensuring patient safety remains equally critical [8].

In Brazil, PBM has gained increasing attention within the medical community; however, comprehensive national data regarding its implementation in cardiovascular surgery remain limited. We hypothesized that PBM knowledge and implementation among Brazilian cardiovascular surgeons would be heterogeneous and associated with regional and institutional factors, as well as perceived implementation barriers. This study aims to evaluate PBM knowledge, implementation, and reported practices among Brazilian cardiovascular surgeons, identify perceived challenges, and provide exploratory data to inform future structured assessments.

## Methods

This cross-sectional, descriptive survey adhered to the ethical and legal standards established by Resolution No. 466/2012 of the Brazilian National Health Council and the guidelines of the International Council on Harmonisation of Technical Requirements for Registration of Pharmaceuticals for Human Use (ICH). The protocol was approved by the Research Ethics Committee of the institution (No. 6.752.162; CAAE: 78128824.2.0000.0068), and data confidentiality was ensured in accordance with the Brazilian General Data Protection Law.

A survey was distributed to 1,105 cardiovascular surgeons registered with the Brazilian Society of Cardiovascular Surgery (SBCCV) to evaluate knowledge and reported practices related to PBM.

Of the 1,105 registered surgeons, 1,058 had valid email addresses and were invited to participate.

A 30-item questionnaire was adapted from a previously published instrument by Frietsch et al. [9] and included items addressing demographic and professional characteristics, preoperative anemia management, transfusion practices, coagulation management, and PBM implementation and barriers (Table 1). The questionnaire was distributed via email on three occasions between August and December 2024. Only one response per participant was accepted. Respondents were instructed to base their answers on the institution with which they had the strongest professional affiliation and to consider their primary institutional practice, including elective and emergency procedures as well as adult and pediatric cardiovascular surgeries.

**Table 1:** Survey - "situational diagnosis of patient blood management (PBM) in cardiological care in Brazil".

Q1. What is your self-identified gender?
Q2. What is your age group?
Q3. What is highest level of education you have completed?
Q4. In your current professional practice, what is the primary area of your surgical activity?
Q5. In which sector do you practice medicine: public sector, private sector, or both?
Q6. In which Brazilian state is your institution located?
Q7. In which Brazilian city is your institution located?
Q8. Approximately how many beds does your institution have?
Q9. What is the average annual volume of cardiac surgeries performed at your institution?
Q10. What is the average prevalence of preoperative anemia among patients in your institution? *Hemoglobin (Hb) <12g/dL in women and Hb <13g/dL in men.
Q11. What is the time frame for preoperative hemoglobin screening in your institution?
Q12. Where are preoperative exams usually conducted in your patients?
Q13. Are all preoperative exams available in the electronic medical records at your institution?
Q14. If anemia is suspected during the preoperative period, which additional tests are usually requested?
Q15. Does your institution provide follow-up and treatment for patients with preoperative anemia?
Q16. How is the preoperative anemia managed in your institution?
Q17. Do you assess erythrocyte mass and blood volume in your patients prior to surgery?
Q18. How do you evaluate the risk of transfusion, blood loss, and the required number of transfusion units?
Q19. Is a hemostasis or hemotherapy specialist available at your institution?
Q20. Which coagulation management tools are routinely used at your institution?
Q21. How often are two or more units of red blood cell concentrates requested for elective procedures?
Q22. Does your institution have a standardized guideline for requesting blood components based on specialty and/or surgical procedure?
Q23. Are the indications for blood transfusion properly documented at your institution?
Q24. How frequently does the transfusion committee at your institution convene?
Q25. Do you have knowledge of PBM principles and practices?
Q26. What clinical blood management practices are implemented at your institution?
Q27. Is your institution interested in implementing PBM?
Q28. What are the main challenges in implementing and managing PBM at your institution?
Q29. Which PBM practices are implemented for elective patients at your institution?
Q30. Which PBM practices are implemented for emergency patients at your institution?

Note: The questionnaire comprised primarily multiple-choice questions, with questions 7, 14, 18, and 28–30 being open-ended narrative questions.

The questionnaire was reviewed by specialists in cardiovascular surgery and hemotherapy to ensure content adequacy prior to dissemination. However, formal psychometric validation, including construct validity and reliability testing, was not performed.

Data were collected using Google Forms® and exported to Microsoft Excel (version 16.16.4) for organization and subsequent statistical analysis. Descriptive statistics included categorical variables expressed as absolute frequency (n) and relative percentage (%). Associations between categorical variables were assessed using Pearson’s chi-square test (χ²) or Fisher’s exact test, with Monte Carlo simulation when appropriate. When statistically significant associations were identified (p-value <0.05), effect size was calculated using Cramér’s V to estimate the magnitude of the observed association. Statistical analyses were conducted using JAMOVI statistical software version 2.2.5® and RStudio version 2024.04.2+764.

## Results

### Demographic and professional profile of cardiovascular surgeons

**Figure 1:**
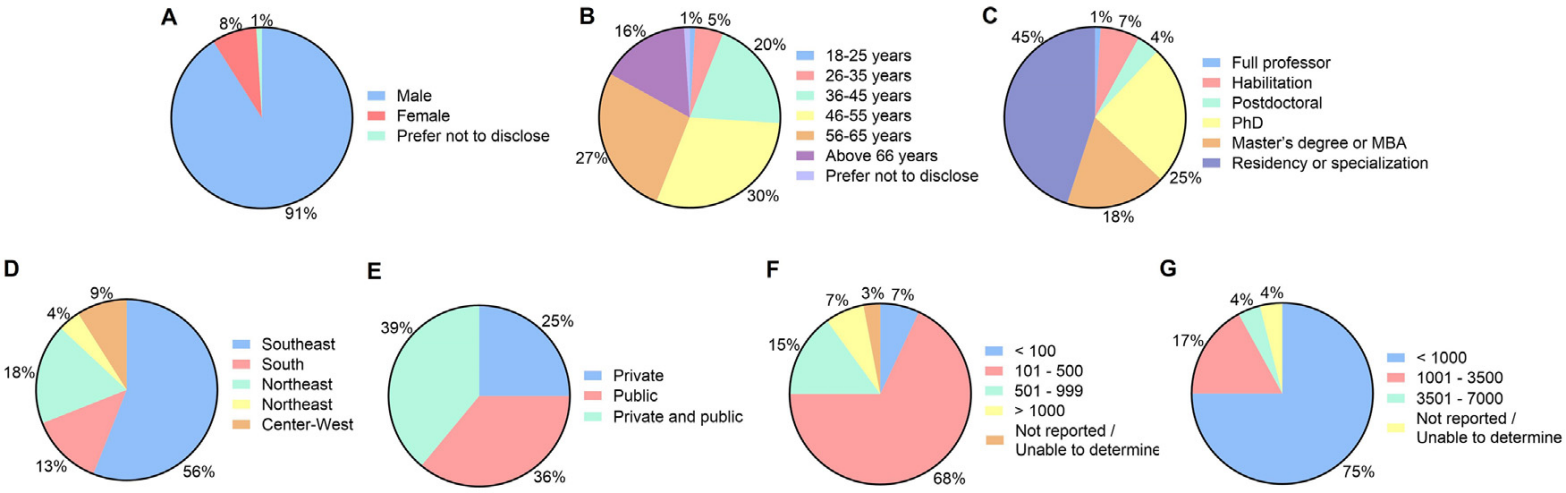
Demographic and professional profile of cardiovascular surgeons. (A) Gender distribution. (B) Age groups. (C) Highest level of academic education. (D) Geographic distribution by Brazilian region. (E) Type of hospital affiliation (public, private, or both). (F) Number of hospital beds. (G) Annual volume of cardiovascular surgeries. (H) Type of cardiovascular surgical practice (adult, pediatric, or both). All percentages are based on respondents (n = 205), unless otherwise specified. Percentages may not total 100% due to rounding.

The overall response rate was 19.4%, corresponding to 205 participants out of 1,058 valid email addresses of SBCCV members. Forty-seven registered members did not receive the survey due to invalid email addresses. The respondents were predominantly male (91%) (Figure 1A), most frequently aged between 46 and 55 years (Figure 1B). The highest level of education reported was residency or specialization (45%) (Figure 1C). Most respondents were affiliated with hospitals located in the southeastern region of Brazil (56%) (Figure 1D), particularly in São Paulo (38%), Minas Gerais (8%), and Rio de Janeiro (7%). No responses were received from surgeons located in the northern states of Amazonas, Roraima, and Acre, or from Sergipe in the northeastern region. A substantial proportion of respondents reported practicing in both the public and private sectors (39%) or exclusively in the public sector (36%) (Figure 1E). Regarding hospital size, 68% indicated that their institutions had between 101 and 500 beds (Figure 1F), and 75% reported performing fewer than 1,000 cardiac surgeries annually (Figure 1G). Additionally, 84% of respondents indicated that their primary surgical practice involved adult cardiovascular surgery.

### Preoperative anemia management and screening protocols

**Figure 2:**
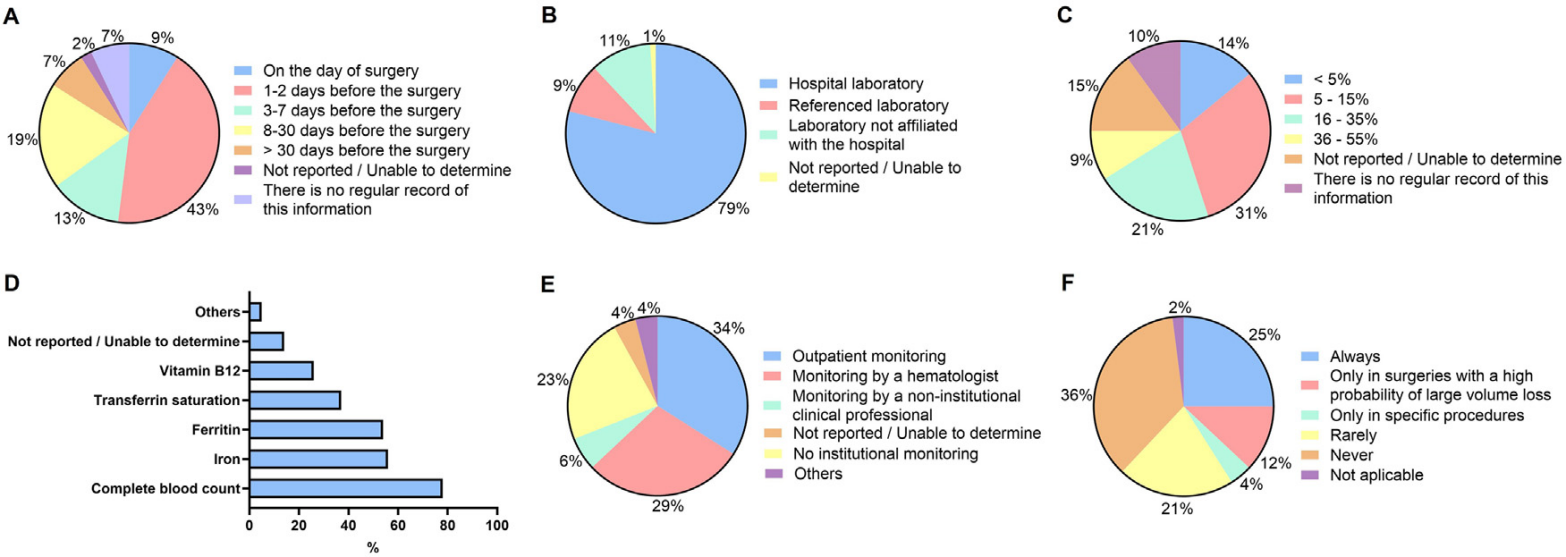
Preoperative anemia screening practices. (A) Timing of hemoglobin testing before surgery. (B) Location where the preoperative test is performed. (C) Estimated prevalence of preoperative anemia among surgical patients. (D) Laboratory tests used to investigate suspected anemia. (E) Settings where anemia treatment is conducted. (F) Frequency of erythrocyte mass and blood volume calculations. Preoperative anemia was defined as hemoglobin <12 g/dL in women and <13 g/dL in men. All percentages are based on respondents (n = 205), unless otherwise specified.

The survey assessed strategies related to preoperative anemia screening and perioperative blood management. Thirty-one percent of respondents estimated the prevalence of preoperative anemia in their institutions to range between 5% and 15% (Figure 2C).

Among patients with suspected anemia, 43% of respondents reported measuring hemoglobin levels 1-2 days prior to surgery (Figure 2A). Additional laboratory tests were commonly requested for diagnostic evaluations and therapeutic planning. A complete blood count was the most frequently requested test (78%), followed by serum iron (56%) and ferritin (54%) (Figure 2D).

Sixty-nine percent of respondents indicated that their institutions provided treatment and follow-up for preoperative anemia, most commonly on an outpatient basis (34%) (Figure 2E). Approximately 25% of respondents reported routinely calculating erythrocyte mass and blood volume prior to surgical procedures (Figure 2F).

### Perioperative blood transfusion and coagulation management

**Figure 3:**
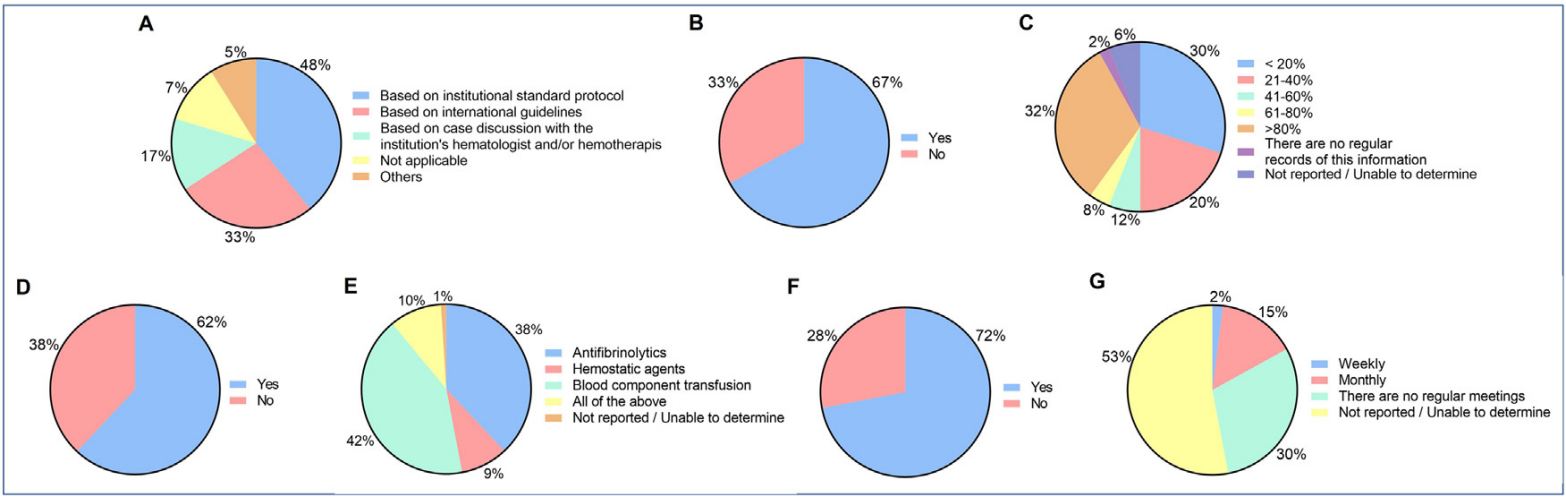
Blood transfusion and coagulation management practices. (A) Existence of institutional protocols for transfusion risk and perioperative blood loss. (B) Availability of hemostasis/hemotherapy professionals for surgical support. (C) Frequency of red blood cell requests for elective procedures. (D) Use of standardized parameters for blood component requests. (E) Most commonly used tools for coagulation management. (F) Documentation of blood transfusion indications. (G) Frequency of transfusion committee meetings. All percentages are based on respondents (n = 205), unless otherwise specified. For multiple-response questions, percentages reflect the proportion of respondents selecting each option. Percentages may not total 100% due to rounding.

Forty-eight percent of respondents reported that their institutions followed a standardized protocol for transfusion risk assessment and perioperative blood loss management (Figure 3A). Sixty-seven percent reported the availability of a hemostasis or hemotherapy specialist for perioperative support (Figure 3B).

The frequency of requesting two or more units of red blood cell concentrates for elective procedures varied: 32% of respondents reported ordering them in more than 80% of surgeries, whereas 30% reported doing so in fewer than 20% of cases (Figure 3C). Additionally, 62% indicated that their institutions used standardized criteria for blood component requests based on specialty or procedure (Figure 3D).

Regarding coagulation management strategies, 42% of respondents reported the use of blood component transfusions, and 38% reported the use of antifibrinolytic agents (Figure 3E). Seventy-two percent reported that indications for blood transfusion were documented in their institutions (Figure 3F).

The frequency of transfusion committee meetings varied across institutions: 30% reported no regular meetings, 15% reported monthly meetings, and 2% reported weekly meetings (Figure 3G).

### Adoption and challenges of patient blood management practices in Brazilian institutions from the respondents’ perspective

**Figure 4:**
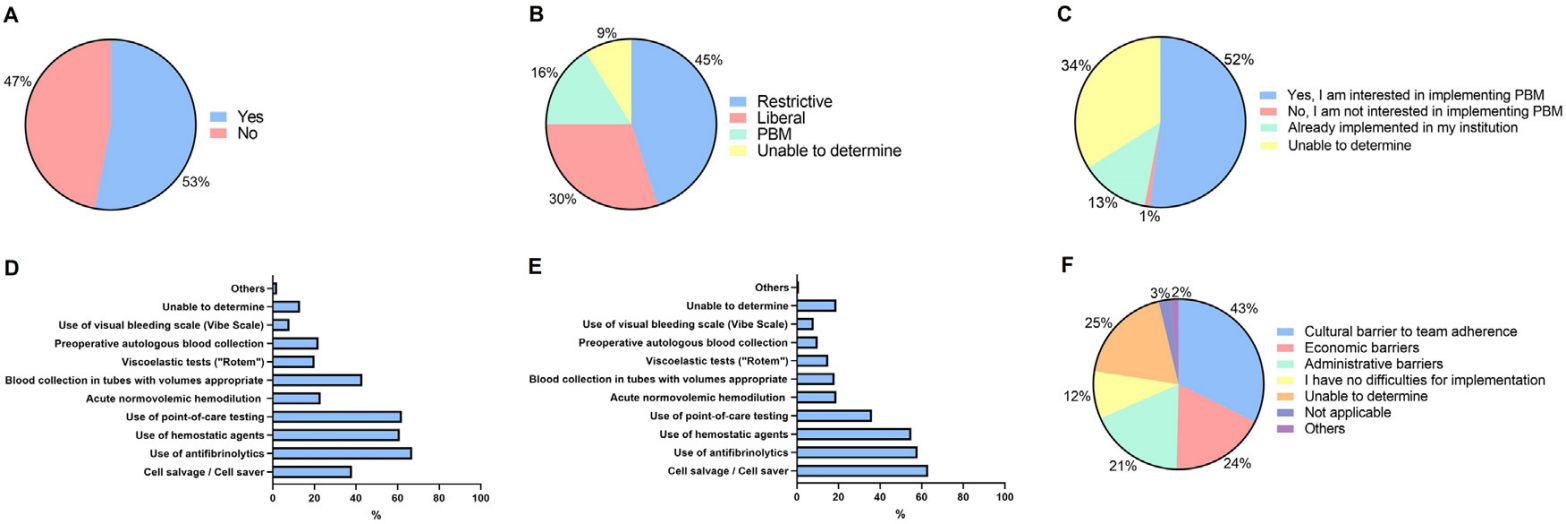
Surgeons’ knowledge, practices, and perceptions regarding patient blood management. (A) Self-reported familiarity with patient blood management (PBM). (B) Transfusion strategies adopted in clinical practice. (C) Surgeons’ interest in implementing PBM in their institutions. (D) PBM interventions most commonly used in elective procedures. (E) PBM strategies used in emergency settings. (F) Main reported barriers to PBM implementation. All percentages are based on respondents (n = 205), unless otherwise specified.

Fifty-three percent of respondents reported being familiar with the concept of PBM (Figure 4A). The most commonly reported transfusion management strategy was the restrictive approach (45%), followed by the liberal approach (30%). Sixteen percent of respondents indicated that PBM guidelines were incorporated into routine clinical practice at their institutions (Figure 4B).

Regarding willingness to adopt PBM practices, 52% of respondents expressed interest in implementing PBM in their institutions, 1% reported no interest, and 13% stated that PBM protocols had already been adopted (Figure 4C).

**Figure 5:**
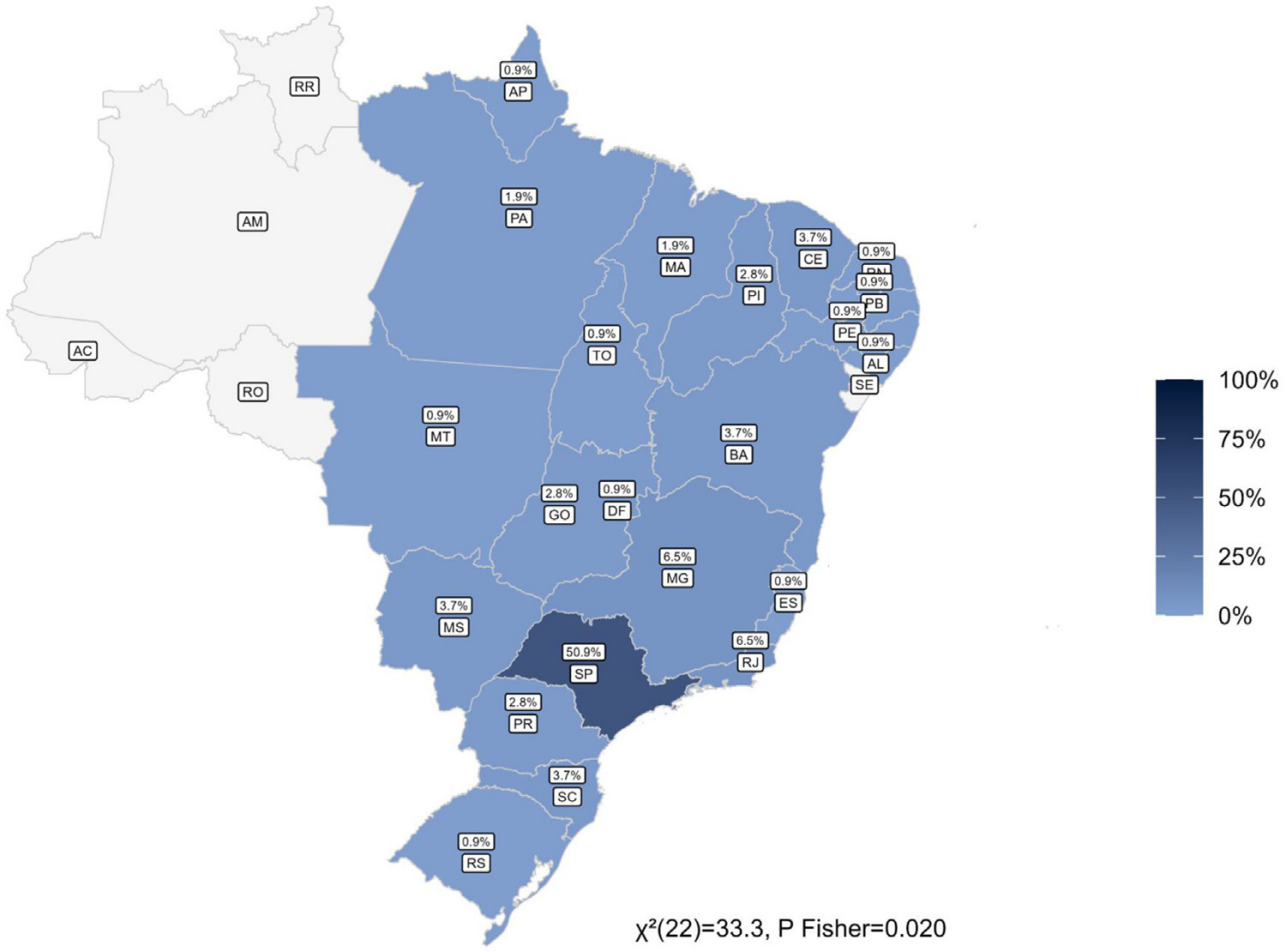
Geographic distribution of respondents interested in implementing patient blood management. Map of Brazil indicating the percentage of surgeons expressing interest in adopting PBM by state. All percentages are based on respondents (n = 205), unless otherwise specified.

Figure 5 illustrates the geographic distribution of respondents who expressed interest in implementing PBM. Among these respondents, 50.9% were based in the state of São Paulo. Reported interest in PBM implementation across other states ranged from 0.9% to 6.5%, with no respondents expressing interest in certain regions. A statistically significant association was observed between geographic region and reported interest in PBM implementation (chi-square (22) = 33.3; p-value = 0.020). The effect size, calculated using Cramér’s V, was 0.403, indicating a moderate association.

Among respondents reporting PBM implementation, the most frequently utilized interventions in elective surgical patients included antifibrinolytics (63%), hemostatic agents (61%), and point-of-care testing (31%) (Figure 4D). Strategies reported in emergency settings included antifibrinolytics (57%), hemostatic agents (58%), and intraoperative blood salvage (55%) (Figure 4E).

The most frequently reported barriers to PBM implementation were cultural resistance (43%), financial constraints (24%), and administrative challenges (21%) (Figure 4F).

## Discussion

The present study provides a cross-sectional situational assessment of knowledge on PBM, reported practices, and perceived barriers among Brazilian cardiovascular surgeons. By exploring surgeons’ perspectives, the findings offer exploratory insights into the heterogeneity of perioperative blood management strategies and the institutional variability surrounding PBM implementation.

The geographic distribution of responses demonstrated greater representation from the southeastern region, particularly São Paulo, while certain northern states and parts of the Northeast were not represented in the sample. Although this may reflect the concentration of high-volume cardiovascular centers and specialized infrastructure in more developed regions, it also underscores the uneven distribution of specialized surgical services across Brazil [10,11]. Importantly, because participation was voluntary and response rates varied regionally, these findings should be interpreted as reflective of the responding cohort rather than definitive national patterns. Nevertheless, regional variations in reported engagement with PBM may suggest differences in access to infrastructure, continuing education opportunities, and institutional support mechanisms.

A central finding of this study was the heterogeneity in the reported management of preoperative anemia. Although anemia is widely recognized as an independent risk factor for perioperative transfusion, postoperative complications, prolonged hospitalization, and mortality [12], structured evaluation and optimization pathways were inconsistently reported. A substantial proportion of respondents indicated that hemoglobin testing was performed only 1–2 days prior to surgery, which significantly limits the therapeutic window for effective intervention. From a clinical standpoint, early identification of anemia, ideally several weeks prior to elective procedures, is essential to allow adequate diagnostic clarification and targeted treatment.

Furthermore, although the complete blood count, serum iron, and ferritin were commonly requested, the reliance on isolated ferritin measurements may be insufficient in cardiac surgical populations. Anemia of inflammation and functional iron deficiency are highly prevalent among patients with chronic cardiovascular disease [13,14]. In such contexts, ferritin levels may be normal or elevated despite depleted bioavailable iron. A comprehensive diagnostic assessment, including transferrin saturation and evaluation of inflammatory markers, is often necessary to differentiate absolute iron deficiency from anemia of chronic disease. Without adequate diagnostic stratification and sufficient preoperative optimization time, opportunities for targeted interventions such as intravenous iron supplementation may be missed, potentially limiting the effectiveness of PBM strategies.

Transfusion practices also demonstrated variability among participating institutions. Approximately half of respondents reported the existence of institutional protocols for transfusion risk assessment and perioperative blood loss management. Although a majority indicated the availability of hemostasis or hemotherapy specialists, not all institutions reported regular transfusion committee meetings or consistent documentation practices. Structured transfusion oversight and institutional protocols have been associated with improved compliance with evidence-based transfusion thresholds and reduced variability in clinical decision-making [15,16]. The variability observed in this survey suggests differing levels of organizational maturity in transfusion governance across the institutions represented in the sample.

Regarding PBM awareness and implementation, slightly more than half of respondents reported familiarity with the PBM concept, yet only a minority indicated formal integration of PBM guidelines into routine institutional practice. This discrepancy between conceptual awareness and structured implementation is consistent with international experiences, where knowledge dissemination alone is insufficient to ensure systemic adoption [17,18]. The most frequently reported barriers (cultural resistance, financial constraints, and administrative challenges) mirror those described in other healthcare systems undergoing transition toward PBM-based models.

The statistically significant association between geographic region and reported interest in PBM implementation further supports the presence of regional variation in engagement. However, this association must be interpreted cautiously, given the response distribution and the cross-sectional design. The findings reflect reported interest rather than objectively verified implementation levels.

In the global context, structured PBM programs implemented in the United States and several European countries have been associated with reductions in transfusion rates, improved adherence to restrictive transfusion strategies, decreased postoperative complications, and cost savings [19,20]. In countries such as Canada and Australia, governmental policies and institutional incentives have facilitated broader adoption of PBM frameworks, including reimbursement models aligned with evidence-based practices [21,22]. While these international experiences provide a relevant benchmark, the present study did not evaluate clinical outcomes, transfusion rates, or economic impact; therefore, direct extrapolation to the Brazilian context is not possible.

Technological infrastructure may represent a facilitating factor for PBM expansion. The high proportion of respondents reporting availability of electronic health records for preoperative laboratory data suggests potential capacity for protocol integration, automated alerts, and data-driven decision support [23, 24]. Integration of electronic systems with standardized PBM pathways may support compliance monitoring and quality improvement initiatives.

Taken together, these findings provide an exploratory overview of surgeons’ perceptions regarding PBM implementation in Brazilian cardiovascular surgery. Rather than establishing definitive national patterns, the study delineates areas of variability in anemia management, transfusion governance, and institutional readiness. By identifying perceived structural and cultural barriers, this survey offers a foundation for future multicenter evaluations, objective institutional audits, and the development of targeted educational and policy-driven initiatives.

## Limitations

Several limitations must be acknowledged. First, the response rate of 19.4% introduces the possibility of non-response bias. Surgeons with greater interest or engagement in PBM may have been more likely to participate, potentially overestimating reported familiarity and implementation. Second, all data were self-reported and not independently verified at the institutional level, limiting the ability to confirm actual practice patterns. Third, certain geographic regions were underrepresented, restricting national generalizability. Fourth, although the questionnaire was adapted from a previously published instrument and reviewed by experts for content adequacy, it did not undergo formal psychometric validation, such as the assessment of reliability and construct validity. Finally, the cross-sectional design precludes evaluation of temporal changes, causal relationships, or outcome associations.

Despite these limitations, this study provides exploratory baseline data regarding surgeons’ perceptions of PBM implementation in Brazilian cardiovascular surgery. The findings highlight areas of variability in anemia management, transfusion governance, and institutional readiness, which may inform future structured national assessments and targeted educational initiatives.

## Conclusion

Among participating cardiovascular surgeons, PBM implementation was reported as heterogeneous across institutions. Respondents identified perceived cultural, economic, and structural barriers to implementation, in addition to variability in anemia management, transfusion governance, and institutional readiness for structured PBM integration. Variability was observed in preoperative anemia management, transfusion governance, and institutional readiness for structured PBM integration. These findings provide exploratory baseline data regarding surgeons’ perceptions and may inform future multicenter evaluations, objective institutional assessments, and targeted educational initiatives. Interpretations should remain within the context of a perception-based cross-sectional survey with limited regional representativeness.

## Funding

CSL Vifor Brazil.

## Conflicts of interest

The authors declare no conflicts of interest.

## Data Availability

The data that support the findings of this study are available from the corresponding author upon reasonable request.
